# Intensifying Functional Task Practice to Meet Aerobic Training Guidelines in Stroke Survivors

**DOI:** 10.3389/fphys.2017.00809

**Published:** 2017-10-26

**Authors:** Liam P. Kelly, Augustine J. Devasahayam, Arthur R. Chaves, Elizabeth M. Wallack, Jason McCarthy, Fabien A. Basset, Michelle Ploughman

**Affiliations:** ^1^Recovery and Performance Lab, Faculty of Medicine, L.A. Miller Centre, Memorial University of Newfoundland, St. John's, NL, Canada; ^2^School of Human Kinetics and Recreation, Memorial University of Newfoundland, St. John's, NL, Canada

**Keywords:** stroke rehabilitation, physical exertion, physical therapy modalities, aerobic exercise, cardiometabolic stress

## Abstract

**Objective:** To determine whether stroke survivors could maintain workloads during functional task practice that can reach moderate levels of cardiometabolic stress (i.e., ≥40% oxygen uptake reserve ($\dot{\text{V}}$O_2_R) for ≥20 min) without the use of ergometer-based exercise.

**Design:** Cross-sectional study using convenience sampling.

**Setting:** Research laboratory in a tertiary rehabilitation hospital.

**Participants:** Chronic hemiparetic stroke survivors (>6-months) who could provide consent and walk with or without assistance.

**Intervention:** A single bout of intermittent functional training (IFT). The IFT protocol lasted 30 min and involved performing impairment specific multi-joint task-oriented movements structured into circuits lasting ~3 min and allowing 30–45 s recovery between circuits. The aim was to achieve an average heart rate (HR) 30-50 beats above resting without using traditional ergometer-based aerobic exercise.

**Outcome measures:** Attainment of indicators for moderate intensity aerobic exercise. Oxygen uptake ($\dot{\text{V}}$O_2_), carbon dioxide production ($\dot{\text{V}}$CO_2_), and HR were recorded throughout the 30 min IFT protocol. Values were reported as percentage of $\dot{\text{V}}$O_2_R, HR reserve (HRR) and HRR calculated from predicted maximum HR (HRR_pred_), which were determined from a prior maximal graded exercise test.

**Results:** Ten (3-female) chronic (38 ± 33 months) stroke survivors (70% ischemic) with significant residual impairments (NIHSS: 3 ± 2) and a high prevalence of comorbid conditions (80% ≥ 1) participated. IFT significantly increased all measures of exercise intensity compared to resting levels: $\dot{\text{V}}$O_2_ (Δ 820 ± 290 ml min^−1^, *p* < 0.001), HR (Δ 42 ± 14 bpm, *p* < 0.001), and energy expenditure (EE; Δ 4.0 ± 1.4 kcal min^−1^, *p* < 0.001). Also, mean values for percentage of $\dot{\text{V}}$O_2_R (62 ± 19), HRR (55 ± 14), and HRR_pred_ (52 ± 18) were significantly higher than the minimum threshold (40%) indicating achievement of moderate intensity aerobic exercise (*p* = 0.004, 0.016, and 0.043, respectively).

**Conclusion:** Sufficient workloads to achieve moderate levels of cardiometabolic stress can be maintained in chronic stroke survivors using impairment-focused functional movements that are not dependent on ergometers or other specialized equipment.

## Introduction

Stroke mortality rate continues to decrease thanks to advances in medical management (Thrift et al., 2017) and emergency medical care (Crichton et al., 2016). However, the amount of recovery observed after disabling stroke remains largely unchanged and the number of individuals living with life-altering physical and cognitive impairments due to stroke is increasing (Krueger et al., 2015). A major challenge to regaining function after disabling stroke is the limited time window for optimal recovery, which is thought to occur within the first 3 months (Cramer, 2008; Murphy and Corbett, 2009). This sensitive period for enhanced plasticity and subsequent plateau of recovery follows well-defined neurobiological processes that involve upregulation of growth-promoting factors followed by their downregulation with concurrent increases in growth-inhibiting factors (Murphy and Corbett, 2009). Substantial resources are currently being employed to develop interventions that extend this time window and possibly even enhance repair mechanisms through use of stem cells, brain stimulation, and other pharmaceutical therapies (Ward, 2017). However, it is unclear whether the plateau of recovery observed post-stroke is due to a failure of the mechanisms underlying spontaneous biological recovery or if it is related to suboptimal dosage of physical and behavioral therapies (Ward, 2017). Animal models of stroke reinforce the critical importance of intense therapeutic exercise combined with an enriched environment to optimize the efficacy of pharmaceutical, and stem cell interventions (Johansson, 2000; Hicks et al., 2009; Ploughman et al., 2009; Sale et al., 2014). Therefore, stroke rehabilitation must be optimized to not only take advantage of intrinsic mechanisms for recovery but also to enhance the effects of emerging therapies.

Unfortunately, current inpatient stroke rehabilitation is of insufficient intensity to promote optimal recovery. Studies from across the globe consistently report that stroke patients spend most of their time “inactive and alone” (Bernhardt et al., 2004) throughout the acute and subacute phases of recovery (Astrand et al., 2016). Also, the total amount of work performed during structured therapy is below levels required to maintain functional fitness (Macko et al., 2005) and reduce cardiovascular risk (MacKay-Lyons and Makrides, 2002). Accordingly, patients in both the acute and chronic phases of recovery demonstrate levels of cardiorespiratory fitness that are about half (~16 ml min^−1^ kg ^−1^) of those observed in age and gender matched populations (Potempa et al., 1995; Smith et al., 2012; Mackay-Lyons et al., 2013a; Ivey et al., 2015). In addition to increasing risk for recurrent stroke (Mackay-Lyons et al., 2013b), such levels of physical deconditioning limit patients' ability to participate in structured therapy (Tang et al., 2009; Billinger et al., 2015) and may even contribute to a ceiling for neuromotor recovery (Ploughman and Kelly, 2016). Given the inverse association between cardiorespiratory fitness and stroke risk (Pandey et al., 2016), premorbid physical activity levels also contribute to the poor aerobic capacity observed after rehabilitation. Regardless, the low intensity nature of the inpatient environment must be addressed to optimize recovery.

Several studies have proposed adding ergometer-based aerobic training to inpatient rehabilitation (Tang et al., 2009; Mackay-Lyons et al., 2013a; Wang et al., 2014), while others have suggested that practice of gross motor skills could produce a training effect if there was adequate attention to heart rate monitoring (Otterman et al., 2012; van de Port et al., 2012; Marsden et al., 2017). In either case, stroke best practice guidelines have advised therapists to provide aerobic training in addition to a minimum of 3 h per day of skilled task training (Billinger et al., 2014; Hebert et al., 2016). In practice, however, there are many challenges to implementing such recommendations including insufficient time, lack of resources, patient level of impairment, and concern for ongoing cardiovascular risk (Bayley et al., 2012; Biasin et al., 2014; Prout et al., 2016). Rehabilitative strategies that are individualized to level of impairment, addressing multiple targets (i.e., relearning of functional tasks and cardiorespiratory fitness), and which do not rely on specialized equipment are urgently needed. The purpose of the current study was to determine whether functional task practice could be structured in such a way to maintain sufficient workloads to reach moderate levels of cardiometabolic stress (i.e., ≥40% oxygen uptake reserve ($\dot{\text{V}}$O_2_R) for ≥20 min) without the use of ergometers. As a first step, an impairment-based intermittent functional training (IFT) protocol was developed to answer this question among chronic stroke survivors. It was hypothesized that (i) workloads maintained during IFT would cause significant elevations in cardiometabolic responses compared to resting values, and (ii) the increased cardiometabolic demands of IFT would be within a range needed to increase cardiorespiratory fitness (i.e., moderate-to-vigorous intensity).

## Methods

### Participants

Chronic stroke survivors (>6 months post-stroke) were recruited from a discharge registry of a tertiary inpatient rehabilitation center as part of an ongoing clinical trial. The local Health Research Ethics Authority approved the study and participants provided written informed consent. Inclusion criteria were: (i) diagnosis of disabling stroke requiring physical rehabilitation, (ii) able to provide consent, and (iii) able to walk at least with assistance.

### Experimental design

Participants visited the laboratory on two separate occasions, with at least 72-h between sessions to avoid any carryover effect. During the first visit, anthropometrics and stroke characteristics [type of stroke, co-morbid conditions, severity of stroke (National Institutes of Health Stroke Scale^1^), and level of impairment (Chedoke-McMaster Impairment Inventory for Leg and Foot) (Gowland et al., 1993)] were recorded prior to performing a maximal graded exercise test (GXT). The GXT was performed to determine maximal oxygen uptake ($\dot{\text{V}}$O_2max_) and maximum heart rate (HR_max_) as described below. The second session lasted ~50 min and included a 10 min seated rest period, followed by 30 min of IFT (described below), finishing with a 10 min seated recovery period. Oxygen uptake ($\dot{\text{V}}$O_2_), carbon dioxide production ($\dot{\text{V}}$CO_2_), breathing frequency, and tidal volume were recorded breath-by-breath throughout the experimental sessions using a portable metabolic cart (VmaxST, Sensor Medics, FL, USA). Heart rate was collected in line with respirometry data using a chest strap sensor (H10, Polar Electro Inc., NY, USA) wirelessly connected to the portable metabolic cart.

### Graded exercise testing

Prior to performing the GXT, participants were assessed for cardiovascular risk by the unit physician using a standardized medical history form (see Supplementary Material). A physician was available during all GXTs. Participants performed the GXT on either a body weight supported treadmill or total body recumbent stepper (TBRS).

The total body recumbent stepper GXT protocol was adapted from previous work in this population (Billinger et al., 2008). Briefly, after familiarizing participants with the TBRS and adjusting the ergometer for arm and leg length, participants maintained 80 steps per minute (SPM) while the load level was gradually increased to level 3. This workload (~20 W) was maintained for the first 2 min, which was then increased by one load level every 2 min at 80 SPM (~20 W increments) until exhaustion or completion of load level 10. If exhaustion was not reached after load level 10, SPM was increased in increments of 10 SPM every 2 min until exhaustion.

The body weight supported treadmill GXT protocol was based on the AEROBICS guidelines (MacKay-Lyons et al., 2012), which involved: 2 min stages beginning with walking at self-selected speed and 0% treadmill grade for 2 min, followed by a 2.5% increase in grade every 2 min until an incline of 10% was reached and, thereafter, a 0.05 m s^−1^ increase in speed every 2 min, until test termination. A <10% bodyweight support was used during the GXT to prevent falls.

Both exercise tests were terminated using predefined criteria: (i) volitional exhaustion, (ii) no increase in $\dot{\text{V}}$O_2_ or HR despite increases in workload, (iii) inability to maintain workload (iv) signs of excessive fatigue. Achievement of $\dot{\text{V}}$O_2max_ was assessed based on attainment of at least two of the following criteria: (i) a plateau in $\dot{\text{V}}$O_2_ (<80 mL min^−1^) despite increasing workload; (ii) respiratory exchange ratio (RER; $\dot{\text{V}}$CO_2_/VO_2_) above 1.10; and (iii) HR_max_ ±10 beats min^−1^ of predicted maximum HR (HR_pred_) calculated as 206.9 − (0.76 × age) or 164 − (0.7 × age) if prescribed beta blockers (American College of Sports Medicine, 2010).

### Intermittent functional training (IFT)

**Figure 1:**
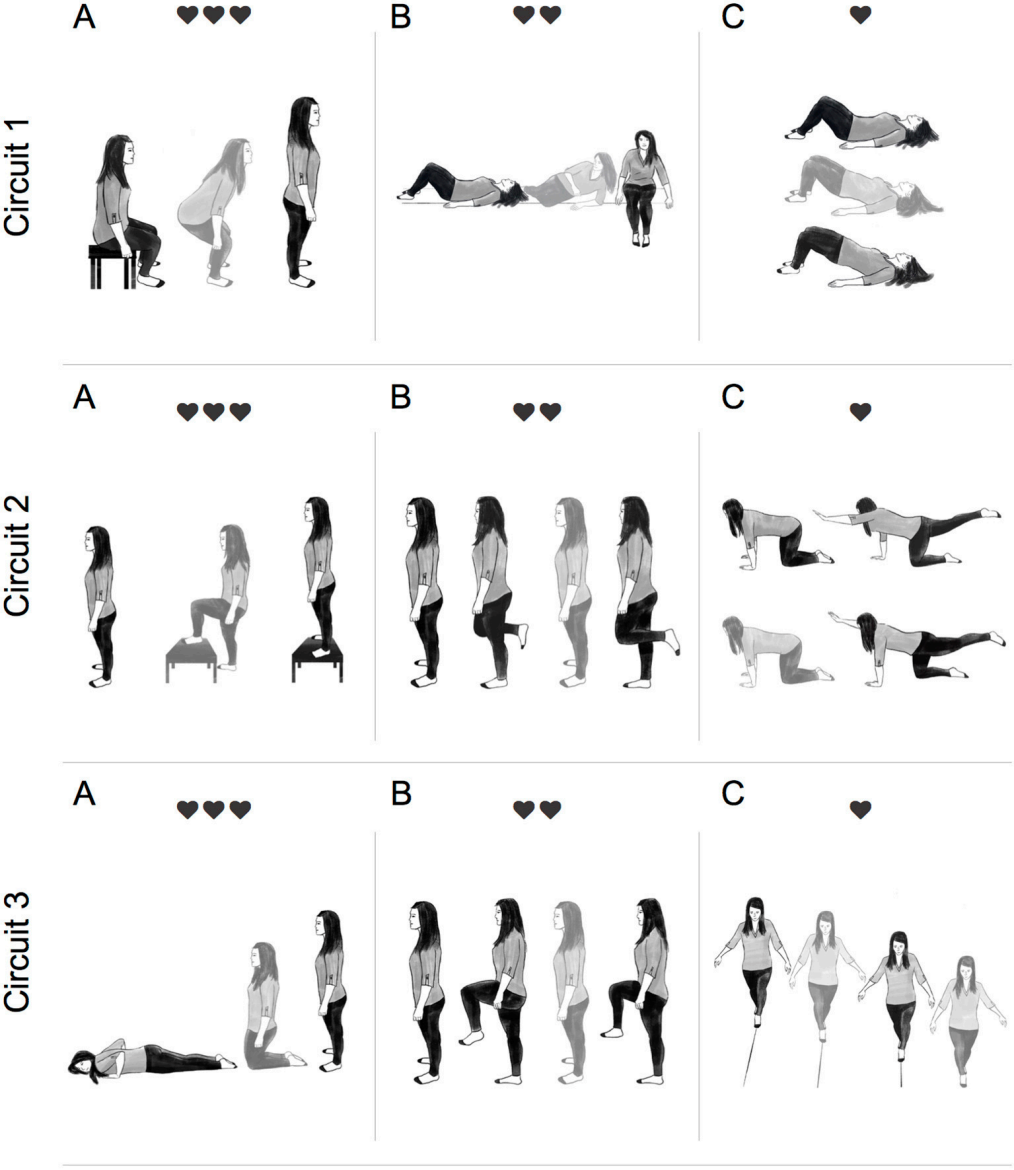
Illustration of a typical intermittent functional training (IFT) session. Participants completed 3 circuits of 3 functional tasks. Each circuit was completed twice, with 30–45 s recovery between sets, before moving onto the next circuit. Circuit 1: **(A)** sit-to-stand (15–20 reps), **(B)** lying-to-sit (7/side), and **(C)** hip bridge (15–20 reps). Circuit 2: **(A)** step-up-to-stand (10–15 reps), **(B)** alternate 1-leg balance (5 sec hold 7/leg), **(C)** alternate-arm and alternate-leg (5 sec hold 7/side). Circuit 3: **(A)** prone-to-standing (15–20 reps), **(B)** high-knees (15–20 reps), **(C)** tandem walking (10 m). Participants were instructed to move from one exercise to the next with minimal rest. ❤ = 15–30 bpm above resting, ❤❤ = 30–45 bpm above resting, and ❤❤❤ = 45–60 bpm above resting.

The IFT protocol involved performing multi-joint task-oriented movements (i.e., sit-to-stand, lying-to-sitting, kneeling-to-stand, etc.) structured into circuits lasting ~3 min and allowing 30–45 s recovery between circuits. Selection of functional tasks were determined based on participant's individual impairments as determined by two registered physiotherapists (MP and AJD). There was 1:1 supervision during IFT and participants were encouraged to minimize rest time within each circuit. The aim was to achieve an average HR 30–50 beats above resting throughout the IFT protocol. As displayed in Figure 1, more metabolically demanding tasks were paired with less demanding ones and consideration was given to minimize task setup and transfer time to maintain the target HR range.

### Calculations

Attainment of the minimum threshold to be considered moderate intensity exercise (American College of Sports Medicine, 2010; MacKay-Lyons et al., 2012) was determined after completion of the IFT protocol. The following equations were used to determine if the minimum threshold criteria (MTC) were exceeded based on 40% of $\dot{\text{V}}$O_2_ reserve ($\dot{\text{V}}$O_2_R), HR reserve (HRR), and HRR calculated from age predicted maximal HR (HRR_pred_):

$$\begin{array}{l} \left(\text{i}\right)\;\dot{\text{V}}O_{2}\text{R}=\left[(\dot{\text{V}}O_{2\text{max}}-\dot{\text{V}}O_{2\text{rest}}{)}^{*}40\%\right]+\dot{\text{V}}O_{2\text{rest}}\\ \left(\text{ii}\right)\text{ HRR}=\left[({\text{HR}}_{\text{max}}-{\text{HR}}_{\text{rest}}{)}^{*}40\%\right]+{\text{HR}}_{\text{rest}}\\ \left(\text{iii}\right)\;{\text{HRR}}_{\text{pred}}=\left[({\text{HR}}_{\text{pred}}-{\text{HR}}_{\text{rest}}{)}^{*}40\%\right]+{\text{HR}}_{\text{rest}} \end{array}$$

Where $\dot{\text{V}}$O_2max_ and HR_max_ were the highest values recorded for oxygen uptake and HR during the GXT, respectively. Resting heart rate (HR_rest_) and oxygen uptake ($\dot{\text{V}}$O_2rest_) were recorded immediately prior to IFT in the seated position. Age-predicted maximal heart rate (HR_pred_) was calculated as described above. Energy expenditure (EE) at rest and during IFT were calculated based on previously published data (Jeukendrup and Wallis, 2005).

### Data reduction and statistical analysis

Heart rate and respirometry data were recorded breath-by-breath and later smoothed using a 30-point box averaging technique. Maximal oxygen uptake was determined during the last 30 s of the GXT and all parameters were reported for the same time point. A mean value representing the 10 min resting periods before and after IFT was reported for all parameters. During IFT both a mean value representing the entire 30 min data collection period and a mean value after removing seated rest time were recorded. Lastly, the cardiometabolic demands of the individual tasks during IFT were reported for the most frequently used tasks.

Due to non-random selection of participants and small sample size, non-parametric statistical tests were used (Graph-Pad Prism, Version 7). Friedman's statistical test was used to detect differences in the cardiometabolic demands at rest, during IFT, and recovery. When appropriate, *a priori post-hoc* analysis were performed using the Dunn's multiple comparisons test. The Wilcoxon signed rank test was used to test for differences in relative exercise intensities based on $\dot{\text{V}}$O_2_R, HRR, and HRR_pred_ compared to the MTC (American College of Sports Medicine, 2010). Data are reported as mean ±SD and statistical significant was set at *p* < 0.05.

## Results

Seven of the 10 participants recruited were male and seven experienced ischemic stroke. As displayed in Table 1, significant residual impairments were observed in participants between 12 and 131 months' post-stroke on both the NIHSS and the Chedoke-McMaster Impairment Inventory (leg & foot). Most participants were overweight, based on the body mass index (BMI), and eight had at least one comorbid condition. Physiological responses to the GXT and MTC are reported in Table 2. All participants could complete the GXT and $\dot{\text{V}}$O_2max_ was between 14 and 29 ml min^−1^ kg^−1^, with a mean value of 20.6 ± 5.4 ml min^−1^ kg^−1^. These values fall within the very poor category according to normative data published by the American College for Sports Medicine (American College of Sports Medicine, 2010). Maximal HR recorded during the GXT was 150 ± 26 bpm, which was within 2% of age-predicted values. Average RER recorded at $\dot{\text{V}}$O_2max_ was 1.06 ± 0.06. The MTC based on 40% of: $\dot{\text{V}}$O_2_R, HRR, and HRR_pred_ were 850 ± 280 ml min^−1^, 103 ± 17 bpm, and 104 ± 14 bpm, respectively.

**Table 1:** Participant characteristics.

**Sub**.	**Age (years)**	**Sex (M,F)**	**Weight (kg)**	**BMI (kg/m^2^)**	**Stroke type**	**Months since stroke**	**NIHSS (/42)**	**Combined Chedoke (/14)**	**Hypertension**	**Diabetes**	**Dyslipidemia**
01	61	M	119	35.9	Ischemic	24	1	13	✔	✔	✔
02	43	M	64	20.4	Ischemic	27	7	4	✔	✘	✔
03	62	M	82	26.2	Ischemic	33	4	7	✘	✘	✘
04	69	M	85	27.2	Ischemic	26	1	13	✘	✔	✘
05	49	F	85	30.5	Hemorrhagic	12	5	9	✔	✘	✘
06	79	F	61	28.0	Ischemic	24	0	12	✘	✘	✘
07	76	M	79	25.1	Ischemic	131	3	11	✘	✔	✘
08	67	M	90	29.8	Ischemic	32	3	12	✘	✔	✔
09	59	F	65	23.0	Hemorrhagic	40	2	9	✔	✘	✘
10	81	M	82	27.4	Hemorrhagic	31	3	12	✔	✘	✔

*NIHSS, National Institute of Health Stroke Scale*.

**Table 2:** Physiological responses recorded during graded exercise test and calculated minimum threshold criteria.

**Sub**.	**Resting HR (min^−1^)**	**Resting $\dot{\text{V}}$O_2_ (ml min^−1^)**	**$\dot{\text{V}}$O_2max_ (ml min^−1^)**	**RER at max**	**$\dot{\text{V}}$O_2max_ (ml min^−1^ kg^−1^)**	**HR_max_ (min^−1^)**	**HR_pred_ (min^−1^)**	**Minimum threshold criteria**		
								**$\dot{\text{V}}$O_2_R (ml min^−1^)**	**HRR (min^−1^)**	**HRR_pred_ (min^−1^)**
01	91	393	2,950	1.01	24.7	150	161	1,415	115	119
02	80	279	1,640	1.08	24.5	185	173	823	122	117
03	66	410	2,570	1.11	29.4	172	160	1,274	108	104
04	80	303	1,760	1.07	20.2	168	154	886	115	110
05	94	320	1,190	1.03	14.0	171	170	668	125	124
06	60	213	840	0.91	13.9	122	147	464	85	95
07	63	124	1,670	1.11	21.5	148	149	742	97	97
08	62	306	1,710	1.13	18.9	138	156	868	92	100
09	61	243	1,670	1.11	25.1	137	162	814	91	101
10	54	462	1,150	1.02	14.1	98	107	737	72	75

*HR, heart rate; $\dot{\text{V}}$O_2_, oxygen uptake; $\dot{\text{V}}$O_2max_, maximal oxygen uptake; RER, respiratory exchange ratio; $\dot{\text{V}}$O_2_R, oxygen uptake reserve; HRR, heart rate reserve*.

**Figure 2:**
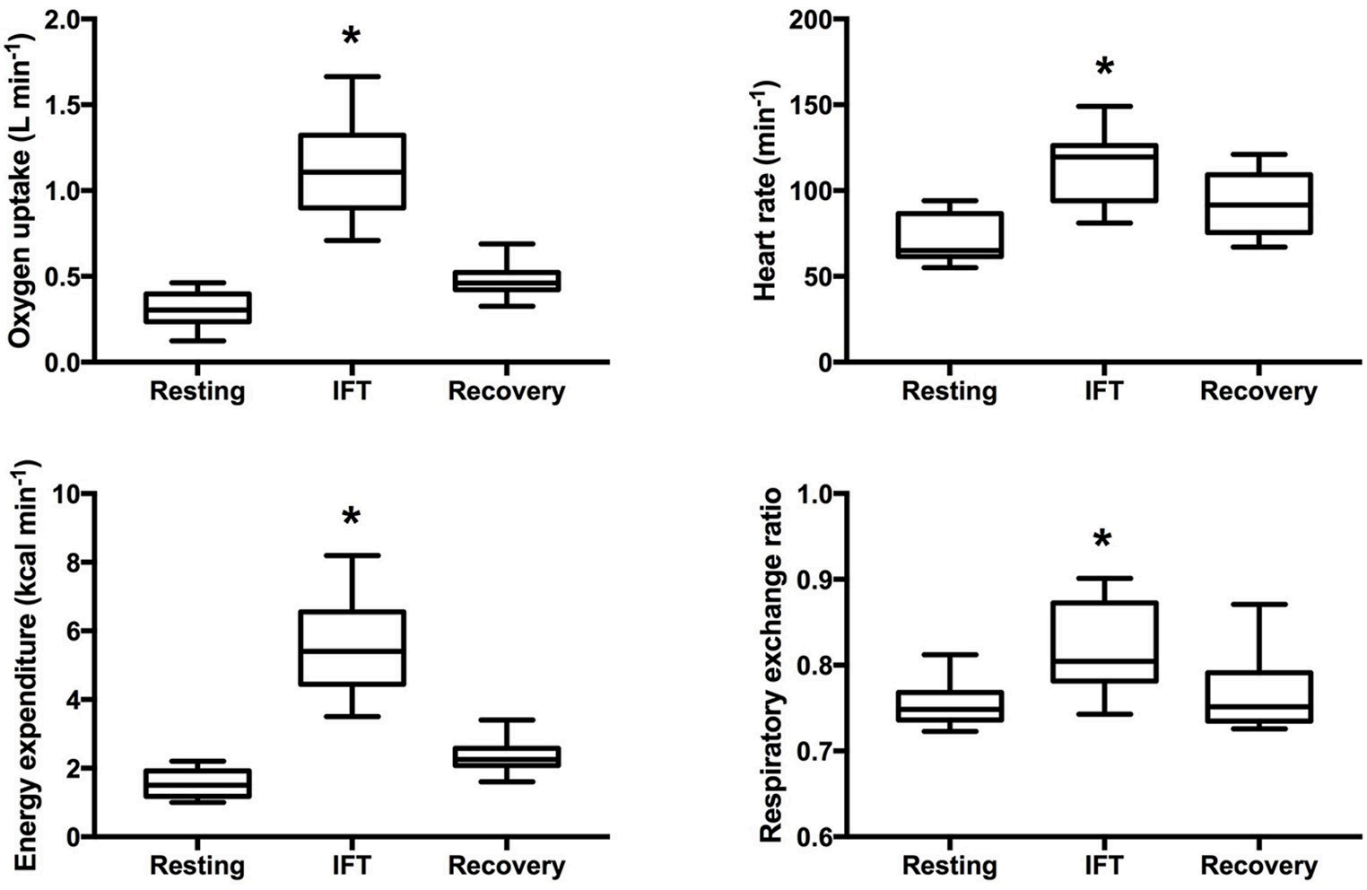
Cardiometabolic responses to intermittent functional training. Box and whisker plot displaying 5th–95th percentile, mean and standard deviation. ^*^Significantly different from pre-exercise resting *p* < 0.05.

Throughout IFT, participants spent more than 80% of the time engaged in task-oriented exercise and less than 5 min was characterized as seated rest time (see Table 3). As displayed in Figure 2, IFT significantly increased all measures of workload compared to resting levels: $\dot{\text{V}}$O_2_ (Δ 820 ± 290 ml min^−1^, *p* < 0.001), HR (Δ 42 ± 14 bpm, *p* < 0.001), EE (Δ 4.0 ± 1.4 kcal min^−1^, *p* < 0.001), and RER (Δ 0.06 ± 0.04, *p* < 0.001). Although not significantly different from pre-exercise values, three of the four measures remained elevated during the 10 min seated post-exercise recovery period: $\dot{\text{V}}$O_2_ (Δ 170 ± 90 ml min^−1^, *p* = 0.051), HR (Δ 22 ± 8 bpm, *p* = 0.051), and EE (Δ 0.8 ± 0.4 kcal min^−1^, *p* = 0.051).

**Table 3:** Participant responses to intermittent functional training excluding rest time.

**Sub**.	**Time (mm:ss)**	**HR (min^−1^)**	**$\dot{\text{V}}$O_2_ (ml min^−1^)**	**MET**	**Achievement of minimum threshold criteria**		
					**HR**	**HR_pred_**	**$\dot{\text{V}}$O_2_**
01	21:05	115	1,664	4.23	Yes	No	Yes
02	23.43	125	954	4.28	Yes	Yes	Yes
03	23:03	109	1,240	4.02	Yes	Yes	No
04	21:11	126	710	2.36	Yes	Yes	No
05	29:23	91	922	4.07	No	No	Yes
06	19:28	149	1,309	4.32	Yes	Yes	Yes
07	25:13	118	1,363	5.00	Yes	Yes	Yes
08	19:58	95	833	3.89	No	Yes	No
09	24:50	121	1,212	3.84	Yes	Yes	Yes
10	19:50	81	1,003	3.50	Yes	Yes	Yes

*IFT, intermittent functional training; HR, heart rate; $\dot{\text{V}}$O_2_, oxygen uptake; MET, metabolic equivalent; yes, achieved criteria; no, did not achieve criteria*.

**Figure 3:**
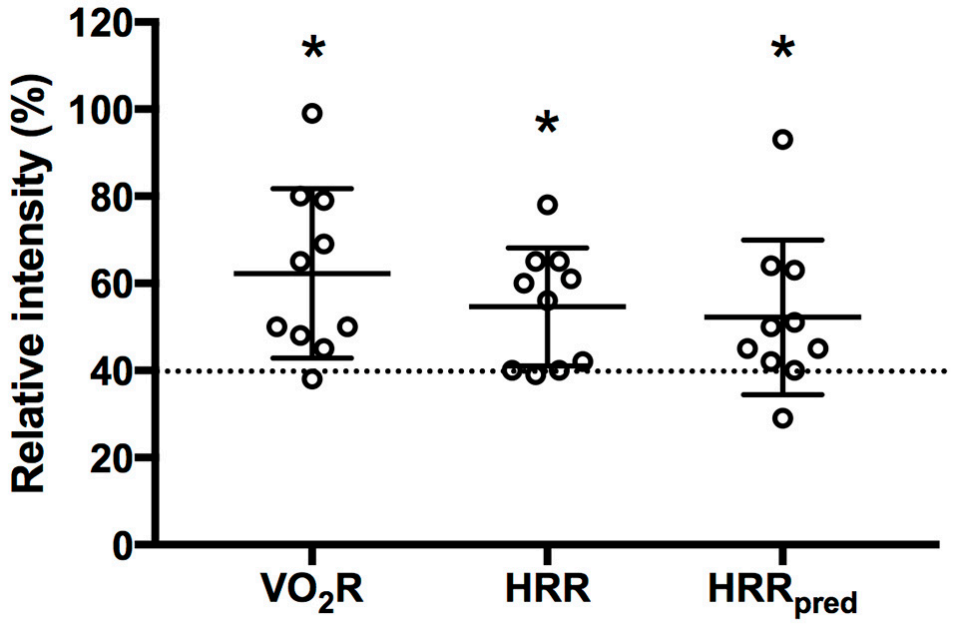
Mean response during intermittent functional training based on oxygen uptake reserve ($\dot{\text{V}}$O_2_R), heart rate reserve (HRR), and HRR using age predicted maximal heart rate (HRR_pred_). Dashed line indicates minimum threshold required to be considered moderate intensity aerobic exercise. ^*^p < 0.05 compared to 40% threshold.

**Figure 4:**
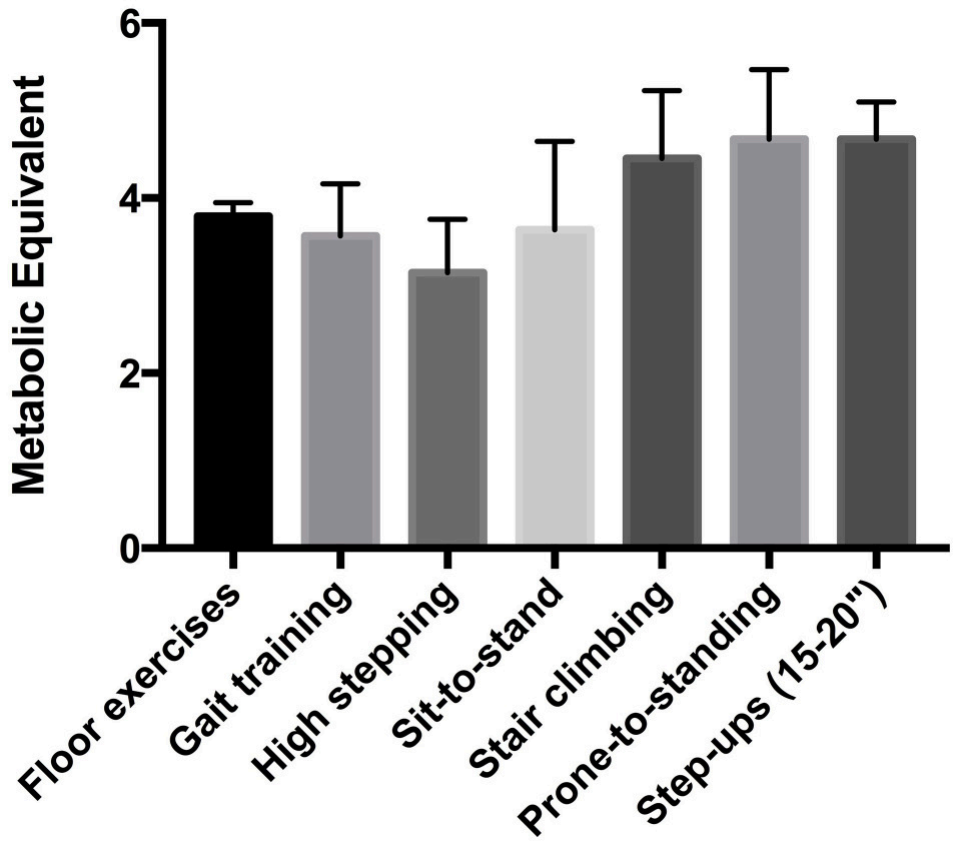
Metabolic equivalent (MET) of functional tasks used during intermittent functional training.

All participants could achieve at least one of the MTC during IFT (Table 3). In fact, mean values for percentage of $\dot{\text{V}}$O_2_R (62 ± 19%), HRR (55 ± 14%), and HRR_pred_ (52 ± 18%) were significantly higher than the minimum threshold (40%) indicating moderate intensity exercise (*p* = 0.004, 0.016, and 0.043, respectively; Figure 3). Also, the metabolic equivalents (MET) for individual task-oriented exercises when completed in a circuit with minimal rest periods were greater than 2.5 (see Figure 4). Practice of standing up from the prone position and stepping-up to stand on 15–20-inch box were the most metabolically challenging tasks at 4–4.5 METs.

## Discussion

We aimed to determine whether practice of individualized functional tasks while keeping HR 30–50 beats above resting for 30 min would be sufficient to achieve the minimum threshold of activity needed to be considered moderate intensity aerobic exercise. Our intention was to develop a method that could be used to enrich stroke rehabilitation by targeting both relearning of neuromotor control and cardiorespiratory fitness. The key finding of the current study was that chronic stroke survivors could sustain workloads during the IFT protocol that were beyond the minimum intensity needed to increase cardiorespiratory fitness (American College of Sports Medicine, 2010) without the use of ergometers or other specialized equipment. All participants reached at least one of the MTC and average HR was between 40 and 80% of HRR. Using similar intermittent training approaches, Marsden et al. (2017) also reported workloads in the moderate to vigorous intensity range using task-oriented exercise in subacute stroke survivors. However, they combined circuits of both functional and ergometer-based exercise to meet aerobic exercise guidelines in this population. Such relative workloads have also been recorded using circuit-based functional movements in populations without acquired brain injury (Paoli et al., 2013; Miller et al., 2014; Schmidt et al., 2016; Sperlich et al., 2017). During high-intensity circuit training using resistance-based exercise, overweight males were able to maintain workloads corresponding to 85% of maximal HR for 30 min 3 times per week over 4 weeks (Miller et al., 2014). Interestingly, this strategy was sufficient to improve blood pressure, glucose tolerance, blood lipids, and body composition (Miller et al., 2014). Although we used tasks that are commonly employed during contemporary stroke rehabilitation, the cardiometabolic demands were much higher than those previously reported (MacKay-Lyons and Makrides, 2002). In the current study, however, more emphasis was placed on the total amount of work performed rather than on the biomechanics and therapists should consider the tradeoff (quality/quantity) before implementing such a program.

The appeal of such intermittent functional training paradigms lies in the fact that it pairs an aerobic exercise stimulus along with task-oriented exercise, which may provide synergetic benefits on ambulatory outcomes in stroke survivors. Outermans et al. (2010), demonstrated that adding 55 min of high intensity task-oriented circuit training to 30 min of usual physiotherapy was more effective than adding the same amount of typical therapy on gait parameters and walking endurance. The safety and efficacy of such therapeutic exercise has also been demonstrated in a relatively large (*n* = 250) multicenter randomized control trial (van de Port et al., 2012). The authors reported significant improvements in walking speed and endurance using task-oriented circuit training in stroke survivors who had completed inpatient therapy. Although these studies were not designed to elucidate the underlying mechanisms, the authors argued that improvements in ambulatory function were likely related to a higher cardiorespiratory workload during circuit training (Outermans et al., 2010). This would then suggest that changes in walking speed and endurance resulted from increased cardiorespiratory fitness rather than improved neuromotor control of gait. However, aerobic exercise not only increases capacity for work but it also stimulates structural and functional alterations in the brain after stroke (Ploughman et al., 2015). It is believed that this beneficial effect is mediated via increased expression of growth factors, such as brain derived neurotrophic factor, which is associated with functional recovery after stroke (Ploughman et al., 2009). It is then conceivable that circuit-based training, which combines task-oriented and aerobic exercise, could enhance neuromotor recovery through synergistic effects on neuroplasticity. This is an important area for future research.

The intermittent functional training protocol described in the current study overcomes three major barriers to implementing aerobic exercise during stroke rehabilitation. Firstly, insufficient time within a single therapy session is consistently reported as a challenge to implementing aerobic exercise recommendations (Biasin et al., 2014; Prout et al., 2016). Therapists have been asked to provide at least 30 min of moderate intensity aerobic exercise three times per week in addition to 3 h per day task specific training (Billinger et al., 2014; Hebert et al., 2016) without corresponding increases in therapy time (Bayley et al., 2012). Although the tradeoff between quantity and quality of task-oriented exercise performed during the IFT protocol needs to be considered, gradually decreasing rest time and organizing tasks to maintain HR 30–50 beats above resting within a therapy session (e.g., the last 20–30 min) is achievable and may be an effective strategy to meet best practice guidelines without increasing therapy time. Secondly, many rehabilitation centers do not have structured aerobic exercise programs and access to appropriate ergometers is a significant barrier to implementation (Prout et al., 2016). Unlike other task-oriented circuit training protocols (Outermans et al., 2010; van de Port et al., 2012; Marsden et al., 2017), the current study was performed without any specialized equipment other than a HR monitor and items which are typically available in stroke rehabilitation units (i.e., floor mat, step, and a bed). Furthermore, the minimal equipment requirement makes the IFT protocol transferrable to the community as part of a home-based exercise program. Thirdly, uncertainty on how to progress patients with ongoing cardiovascular risk and comorbid conditions has been identified as a barrier to reaching target HR training zones (Biasin et al., 2014). This is further complicated by recommendations to perform maximal exercise testing with electrocardiography monitoring (MacKay-Lyons et al., 2012), which is not routinely available in rehabilitation units. Progression within the IFT protocol could be achieved by increasing the complexity of a given functional task (e.g., practice on unstable surface) rather than increasing the target HR. Although IFT may help to address several challenges to implementation of aerobic exercise recommendations, it is still unknown whether such exercise strategies can replicate the demonstrated benefits observed with ergometer-based aerobic exercise on CRF (Mackay-Lyons et al., 2013a; Ivey et al., 2015) and cardiometabolic risk factors (D'Isabella et al., 2017) in stroke survivors. A comparison between the two methods is warranted.

### Study limitations

As a first step toward understanding the potential utility of providing intensive task-oriented exercise during inpatient rehabilitation, we evaluated the cardiometabolic demands of our IFT protocol in chronic stroke survivors. The generalizability of these results to patients at earlier stages into their recovery is a significant limitation. However, it must be realized that tasks were individualized to each participant's level of impairment and such tasks are commonly employed during inpatient rehabilitation. Also, the cardiovascular demands were within the range recommended for patients within the first 3 months (MacKay-Lyons et al., 2012) and did not require the use of specialized equipment.

## Conclusion

The level of physical deconditioning observed in stroke survivors not only increases risk for recurrent stroke and development of comorbid conditions but can also limit participation in rehabilitation programs. Stroke best practice guidelines now encourage incorporating aerobic exercise as soon as possible during inpatient rehabilitation and throughout the continuum of care (Billinger et al., 2014; Hebert et al., 2016). However, there are many challenges to implementing aerobic exercise recommendations during inpatient rehabilitation including access to appropriate ergometers, ability to maintain target heart rate training zone, and assessment of underlying cardiovascular risk. The current analysis revealed that typical functional tasks employed during inpatient rehabilitation can be organized to meet aerobic training guidelines in chronic stroke survivors with significant cardiovascular comorbidities and residual impairments. The advantage of such exercise modalities is that specialized equipment is not required, tasks are individualized to impairments, and progression can be achieved by increasing difficulty of movement rather than only increasing the target HR. When implemented into a progressive training program (Ploughman and Kelly, 2016), IFT has the potential to address multiple targets for regaining function after stroke. More studies are need to describe the cardiometabolic demands in subacute stroke survivors and to determine the efficacy of such training programs on functional recovery, CRF, and cardiovascular risk factors.

## Ethics statement

This study was carried out in accordance with the recommendations of the Tri-Council Policy Statement: Ethical Conduct for Research Involving Humans (TCPS2), Health Research Ethics Board (HREB), with written informed consent from all subjects. All subjects gave written informed consent in accordance with the Declaration of Helsinki. The protocol was approved by the HREB.

## Author contributions

LK conceived of and designed experiment, collected data, analyzed data, interpreted findings, and wrote the manuscript. AD screened subjects, collected the data and edited manuscript. AC conceived of experiment and edited manuscript. EW recruited participants and edited manuscript. JM designed the experiment and screened subjects. FB interpreted findings and edited manuscript. MP conceived of and designed the experiment, screened subjects, and edited manuscript.

### Conflict of interest statement

The authors declare that the research was conducted in the absence of any commercial or financial relationships that could be construed as a potential conflict of interest.

## Footnotes

^1^National Institute of Health Stroke Scale. Available online at: https://www.ninds.nih.gov/sites/default/files/NIH_Stroke_Scale_Booklet.pdf (Accessed July 21 2017).
