# Supplementary material for: Intensifying Functional Task Practice to Meet Aerobic Training Guidelines in Stroke Survivors
Source: Front Physiol. 2017 Oct 26;8:809. doi: 10.3389/fphys.2017.00809 (PMC5662635; doi:10.3389/fphys.2017.00809)
Supplement: Supplementary file 1 [file DataSheet1.PDF]

Physical Activity Readiness
Medical Examination
(revised 2002)

PARmed-X

PHYSICAL ACTIVITY READINESS MEDICAL EXAMINATION

The PARmed-X is a physical activity-specific checklist to be used by a physician with patients who have had positive responses to the Physical Activity Readiness Questionnaire (PAR-Q). In addition, the Conveyance/Referral Form in the PARmed-X can be used to convey clearance for physical activity participation, or to make a referral to a medically-supervised exercise program.

Regular physical activity is fun and healthy, and increasingly more people are starting to become more active every day. Being more active is very safe for most people. The PAR-Q by itself provides adequate screening for the majority of people. However, some individuals may require a medical evaluation and specific advice (exercise prescription) due to one or more positive responses to the PAR-Q.

Following the participant's evaluation by a physician, a physical activity plan should be devised in consultation with a physical activity professional (CSEP-Certified Personal Trainer™ or CSEP-Certified Exercise Physiologist™). To assist in this, the following instructions are provided:

PAGE 1: • Sections A, B, C, and D should be completed by the participant BEFORE the examination by the physician. The bottom section is to be completed by the examining physician.

PAGES 2 & 3: • A checklist of medical conditions requiring special consideration and management.

PAGE 4: • Physical Activity & Lifestyle Advice for people who do not require specific instructions or prescribed exercise.

• Physical Activity Readiness Conveyance/Referral Form - an optional tear-off tab for the physician to convey clearance for physical activity participation, or to make a referral to a medically-supervised exercise program.

This section to be completed by the participant

A PERSONAL INFORMATION:

NAME ______________________________

ADDRESS ______________________________

____

TELEPHONE ______________________________

BIRTHDATE ____________________ GENDER __________

MEDICAL No. ______________________________

B PAR-Q: Please indicate the PAR-Q questions to which you answered YES

- ❑ Q 1 Heart condition
- ❑ Q 2 Chest pain during activity
- ❑ Q 3 Chest pain at rest
- ❑ Q 4 Loss of balance, dizziness
- ❑ Q 5 Bone or joint problem
- ❑ Q 6 Blood pressure or heart drugs
- ❑ Q 7 Other reason: ______________________________

C RISK FACTORS FOR CARDIOVASCULAR DISEASE:

Check all that apply

- ❑ Less than 30 minutes of moderate physical activity most days of the week.
- ❑ Currently smoker (tobacco smoking 1 or more times per week).
- ❑ High blood pressure reported by physician after repeated measurements.
- ❑ High cholesterol level reported by physician.
- ❑ Excessive accumulation of fat around waist.
- ❑ Family history of heart disease.

***Please note:** Many of these risk factors are modifiable. Please refer to page 4 and discuss with your physician.*

D PHYSICAL ACTIVITY INTENTIONS:

What physical activity do you intend to do?

____

____

____

This section to be completed by the examining physician

Physical Exam:

Ht	Wt	BP i) /
		BP ii) /

Conditions limiting physical activity:

- ❑ Cardiovascular
- ❑ Respiratory
- ❑ Other
- ❑ Musculoskeletal
- ❑ Abdominal

Tests required:

- ❑ ECG
- ❑ Exercise Test
- ❑ X-Ray
- ❑ Blood
- ❑ Urinalysis
- ❑ Other

Physical Activity Readiness Conveyance/Referral:

Based upon a current review of health status, I recommend:

Further Information:
- ❑ Attached
- ❑ To be forwarded
- ❑ Available on request

- ❑ No physical activity
- ❑ Only a medically-supervised exercise program until further medical clearance
- ❑ Progressive physical activity:
  - ❑ with avoidance of: ______________________________
  - ❑ with inclusion of: ______________________________
  - ❑ under the supervision of a CSEP-Certified Exercise Physiologist™
- ❑ Unrestricted physical activity–start slowly and build up gradually

CSEP | SCPE THE GOLD STANDARD IN EXERCISE SCIENCE AND PERSONAL TRAINING
© Canadian Society for Exercise Physiology www.csep.ca

Physical Activity Readiness
Medical Examination
(revised 2002)

PARmed-X PHYSICAL ACTIVITY READINESS MEDICAL EXAMINATION

Following is a checklist of medical conditions for which a degree of precaution and/or special advice should be considered for those who answered "YES" to one or more questions on the PAR-Q, and people over the age of 69. Conditions are grouped by system. Three categories of precautions are provided. Comments under Advice are general, since details and alternatives require clinical judgement in each individual instance.

	Absolute Contraindications	Relative Contraindications	Special Prescriptive Conditions	ADVICE
	Permanent restriction or temporary restriction until condition is treated, stable, and/or past acute phase.	Highly variable. Value of exercise testing and/or program may exceed risk. Activity may be restricted.<br>Desirable to maximize control of condition.<br>Direct or indirect medical supervision of exercise program may be desirable.	Individualized prescriptive advice generally appropriate:<br>• limitations imposed; and/or<br>• special exercises prescribed.<br>May require medical monitoring and/or initial supervision in exercise program.	
Cardiovascular	❑ aortic aneurysm (dissecting)<br>❑ aortic stenosis (severe)<br>❑ congestive heart failure<br>❑ crescendo angina<br>❑ myocardial infarction (acute)<br>❑ myocarditis (active or recent)<br>❑ pulmonary or systemic embolism—acute<br>❑ thrombophlebitis<br>❑ ventricular tachycardia and other dangerous dysrhythmias (e.g., multi-focal ventricular activity)	❑ aortic stenosis (moderate)<br>❑ subaortic stenosis (severe)<br>❑ marked cardiac enlargement<br>❑ supraventricular dysrhythmias (uncontrolled or high rate)<br>❑ ventricular ectopic activity (repetitive or frequent)<br>❑ ventricular aneurysm<br>❑ hypertension—untreated or uncontrolled severe (systemic or pulmonary)<br>❑ hypertrophic cardiomyopathy<br>❑ compensated congestive heart failure	❑ aortic (or pulmonary) stenosis—mild angina pectoris and other manifestations of coronary insufficiency (e.g., post-acute infarct)<br>❑ cyanotic heart disease<br>❑ shunts (intermittent or fixed)<br>❑ conduction disturbances<br>• complete AV block<br>• left BBB<br>• Wolff-Parkinson-White syndrome<br>❑ dysrhythmias—controlled<br>❑ fixed rate pacemakers	• clinical exercise test may be warranted in selected cases, for specific determination of functional capacity and limitations and precautions (if any).<br>• slow progression of exercise to levels based on test performance and individual tolerance.<br>• consider individual need for initial conditioning program under medical supervision (indirect or direct).
			❑ intermittent claudication	progressive exercise to tolerance
			❑ hypertension: systolic 160-180; diastolic 105+	progressive exercise; care with medications (serum electrolytes; post-exercise syncope; etc.)
Infections	❑ acute infectious disease (regardless of etiology)	❑ subacute/chronic/recurrent infectious diseases (e.g., malaria, others)	❑ chronic infections<br>❑ HIV	variable as to condition
Metabolic		❑ uncontrolled metabolic disorders (diabetes mellitus, thyrotoxicosis, myxedema)	❑ renal, hepatic & other metabolic insufficiency	variable as to status
			❑ obesity<br>❑ single kidney	dietary moderation, and initial light exercises with slow progression (walking, swimming, cycling)
Pregnancy		❑ complicated pregnancy (e.g., toxemia, hemorrhage, incompetent cervix, etc.)	❑ advanced pregnancy (late 3rd trimester)	refer to the "PARmed-X for PREGNANCY"

References:

Arraix, G.A., Wigle, D.T., Mao, Y. (1992). Risk Assessment of Physical Activity and Physical Fitness in the Canada Health Survey Follow-Up Study. **J. Clin. Epidemiol.** 45:4 419-428.

Mottola, M., Wolfe, L.A. (1994). Active Living and Pregnancy, In: A. Quinney, L. Gauvin, T. Wall (eds.), **Toward Active Living: Proceedings of the International Conference on Physical Activity, Fitness and Health**. Champaign, IL: Human Kinetics.

PAR-Q Validation Report, British Columbia Ministry of Health, 1978.

Thomas, S., Reading, J., Shephard, R.J. (1992). Revision of the Physical Activity Readiness Questionnaire (PAR-Q). **Can. J. Spt. Sci.** 17: 4 338-345.

The PAR-Q and PARmed-X were developed by the British Columbia Ministry of Health. They have been revised by an Expert Advisory Committee of the Canadian Society for Exercise Physiology chaired by Dr. N. Gledhill (2002).

No changes permitted. You are encouraged to photocopy the PARmed-X, but only if you use the entire form.

Disponible en français sous le titre
«Évaluation médicale de l'aptitude à l'activité physique (X-AAP)»

Continued on page 3...

	Special Prescriptive Conditions	ADVICE
Lung	❏ chronic pulmonary disorders	special relaxation and breathing exercises
	❏ obstructive lung disease<br>❏ asthma	breath control during endurance exercises to tolerance; avoid polluted air
	❏ exercise-induced bronchospasm	avoid hyperventilation during exercise; avoid extremely cold conditions; warm up adequately; utilize appropriate medication.
Musculoskeletal	❏ low back conditions (pathological, functional)	avoid or minimize exercise that precipitates or exasperates e.g., forced extreme flexion, extension, and violent twisting; correct posture, proper back exercises
	❏ arthritis—acute (infective, rheumatoid; gout)	treatment, plus judicious blend of rest, splinting and gentle movement
	❏ arthritis—subacute	progressive increase of active exercise therapy
	❏ arthritis—chronic (osteoarthritis and above conditions)	maintenance of mobility and strength; non-weightbearing exercises to minimize joint trauma (e.g., cycling, aquatic activity, etc.)
	❏ orthopaedic	highly variable and individualized
	❏ hernia	minimize straining and isometrics; stregthen abdominal muscles
	❏ osteoporosis or low bone density	avoid exercise with high risk for fracture such as push-ups, curl-ups, vertical jump and trunk forward flexion; engage in low-impact weight-bearing activities and resistance training
CNS	❏ convulsive disorder not completely controlled by medication	minimize or avoid exercise in hazardous environments and/or exercising alone (e.g., swimming, mountain climbing, etc.)
	❏ recent concussion	thorough examination if history of two concussions; review for discontinuation of contact sport if three concussions, depending on duration of unconsciousness, retrograde amnesia, persistent headaches, and other objective evidence of cerebral damage
Blood	❏ anemia—severe (< 10 Gm/dl)<br>❏ electrolyte disturbances	control preferred; exercise as tolerated
Medications	❏ antianginal<br>❏ antihypertensive<br>❏ beta-blockers<br>❏ diuretics<br>❏ others<br>❏ antiarrhythmic<br>❏ anticonvulsant<br>❏ digitalis preparations<br>❏ ganglionic blockers	NOTE: consider underlying condition. Potential for: exertional syncope, electrolyte imbalance, bradycardia, dysrhythmias, impaired coordination and reaction time, heat intolerance. May alter resting and exercise ECG's and exercise test performance.
Other	❏ post-exercise syncope	moderate program
	❏ heat intolerance	prolong cool-down with light activities; avoid exercise in extreme heat
	❏ temporary minor illness	postpone until recovered
	❏ cancer	if potential metastases, test by cycle ergometry, consider non-weight bearing exercises; exercise at lower end of prescriptive range (40-65% of heart rate reserve), depending on condition and recent treatment (radiation, chemotherapy); monitor hemoglobin and lymphocyte counts; add dynamic lifting exercise to strengthen muscles, using machines rather than weights.

*Refer to special publications for elaboration as required

The following companion forms are available online: http://www.csep.ca/forms

The **Physical Activity Readiness Questionnaire (PAR-Q)** - a questionnaire for people aged 15-69 to complete before becoming much more physically active.

The **Physical Activity Readiness Medical Examination for Pregnancy (PARmed-X for PREGNANCY)** - to be used by physicians with pregnant patients who wish to become more physically active.

For more information, please contact the:

Canadian Society for Exercise Physiology
370-18 Louisa Ottawa, ON K1R 6Y6
Tel. 1-877-651-3755 • FAX (613) 234-3565 • Online: www.csep.ca

Note to physical activity professionals...

It is a prudent practice to retain the completed Physical Activity Readiness Conveyance/Referral Form in the participant's file.

CSEP | SCPE
THE GOLD STANDARD IN EXERCISE SCIENCE AND PERSONAL TRAINING

© Canadian Society for Exercise Physiology www.csep.ca/forms

Continued on page 4...

PARmed-X PHYSICAL ACTIVITY READINESS MEDICAL EXAMINATION

PARmed-X Physical Activity Readiness Conveyance/Referral Form

Based upon a current review of the health status of ______________________________, I recommend:

- ❏ No physical activity
- ❏ Only a medically-supervised exercise program until further medical clearance
- ❏ Progressive physical activity
  - ❏ with avoidance of: ______________________________
  - ❏ with inclusion of: ______________________________
  - ❏ under the supervision of a CSEP-Certified Exercise Physiologist™
- ❏ Unrestricted physical activity — start slowly and build up gradually

Further Information:
- ❏ Attached
- ❏ To be forwarded
- ❏ Available on request

Physician/clinic stamp:

______________________________ M.D.

______________________________ 20______
(date)

NOTE: This physical activity clearance is valid for a maximum of six months from the date it is completed and becomes invalid if your medical condition becomes worse.
